# Comparative Molecular Profiling and Bioactivity Analysis of Algerian Propolis: Antioxidant, Antibacterial Activities, and In Silico NRF2-KEAP1 Pathway Modulation

**DOI:** 10.3390/cimb47090761

**Published:** 2025-09-15

**Authors:** Amel Reguig, Ahmed Messai, Ibtissam Kahina Bedaida, Diana C. G. A. Pinto, Chawki Bensouici, Abdelmoneim Tarek Ouamane, Artur M. S. Silva, Jean-Philippe Roy

**Affiliations:** 1 DEDSPAZA Laboratory, Department of Agronomical Sciences, Faculty of Exact Sciences and Natural and Life Sciences, University of Biskra, Biskra 07000, Algeria; amel.reguig@univ-biskra.dz; 2 PIARA Laboratory, Department of Agronomical Sciences, Faculty of Exact Sciences and Natural and Life Sciences, University of Biskra, Biskra 07000, Algeria; 3 Laboratory of Biotechnology of Bioactive Molecules and Cellular Physiopathology (LBMBPC), Faculty of Natural and Life Sciences, University of Batna 2, Batna 05000, Algeria; i.bedaida@univ-batna2.dz; 4 LAQV-REQUIMTE, Department of Chemistry, University of Aveiro, 3810-193 Aveiro, Portugal; 5 National Center for Biotechnological Research (CRBT), BP E73 Ali Mendjili, Constantine 25000, Algeria; 6 Center for Scientific and Technical Research on Arid Regions (CRSTRA), Biskra 07000, Algeria; altarek07@gmail.com; 7 Department of Clinical Sciences, Université de Montréal, Saint-Hyacinthe, QC J2S 2M2, Canada

**Keywords:** bee product, characterization, GC-MS, UHPLC-DAD-ESI/MS, molecular docking, ADME-Tox

## Abstract

Propolis, a natural bee-derived product rich in diverse phytochemicals with potential therapeutic benefits, remains underexplored in Algeria. This study investigated the molecular profile, antioxidant capacity, and antibacterial activity of propolis sourced from two bioclimatically distinct Algerian regions (humid subtropical Batna and hot desert Biskra) using electrospray ionization mass spectrometry, ultra-high-performance liquid chromatography with diode array detection, and gas chromatography–mass spectrometry. Significant regional variations were observed, with propolis extract 2 (PE2) exhibiting a higher bioactive content, including a constituent not previously reported in propolis. Antioxidant assays (2,2-diphenyl-1-picrylhydrazyl, 2,2′-azino-bis (3-ethylbenzothiazoline-6-sulfonic acid), ferric reducing antioxidant power, and phenanthroline) demonstrated that PE2 consistently outperformed propolis extract 1 and the reference standards (DPPH IC_50_: 27.74 µg/mL; FRAP: 5.16 µg/mL). Antibacterial testing demonstrated potent bactericidal effects, particularly for PE2, with minimum inhibitory concentration values equivalent to the minimum bactericidal concentrations required against *Staphylococcus aureus* ATCC 25923 (18.75 µg/mL) and *Escherichia coli* ATCC 25922 (133 µg/mL). Molecular docking identified nine bioactive compounds with high KEAP1 binding affinity, with 1,3-*O*-caffeoyl-dihydrocaffeoylglycerol (first time reported in propolis) showing the strongest binding affinity (−11.02 Kcal/mol). In silico pharmacokinetic predictions further verified its drug-like properties. These findings suggest the tested Algerian propolis samples, as a source of natural alternative antioxidants and antimicrobials, provide a basis for future research in drug discovery and development.

## 1. Introduction

Beekeeping is a multifaceted and beneficial practice that offers numerous advantages to society. By fostering biodiversity through pollination and supporting the sustainable development of rural areas, it plays a vital role in producing various plant-derived food sources crucial for human consumption and food security [1]. This essential sector directly yields high-demand products such as propolis, highly valued for its diverse functions, including significant medicinal and therapeutic properties [2]. Propolis is a complex, natural hive product gathered by *Apis mellifera* bees from various plant sources, such as flowers, trees, and bud secretions [3]. Inside the hive, bees combine these plant resins with their enzymatic secretions, waxes, and pollen [4]. The resulting biochemical composition is remarkably diverse and highly influenced by factors including plant origin, geographic location, season, and even environmental conditions such as solar radiation, precipitation, and humidity [3,5]. For instance, Brazilian propolis alone has been categorized into 12 distinct groups based on established criteria, highlighting this variability [6]. Typically, propolis consists of approximately 50% plant resins, 30% waxes, 10% essential oils, 5% pollen, and 5% various organic compounds, including phenols, terpenes, esters, amino acids, vitamins, minerals, and sugars [4].

Building on its long history of use in folk medicine, modern research has extensively demonstrated the diverse biological properties of propolis, attributed to its rich array of bioactive compounds. These include significant antioxidant [7,8], anti-inflammatory, antibacterial, and anticancer activities [9]. Moreover, besides its medicinal uses, propolis has numerous applications in the food and cosmetic industries [4,10]. Increasingly, propolis’ broad-spectrum biological activities are positioning it as a sustainable alternative to conventional antibiotics in veterinary medicine [11]. This transition is especially urgent for combating antimicrobial resistance (AMR) in high-priority applications in livestock and companion animals, such as bovine mastitis, canine otitis, and poultry health [11].

To date, over 800 distinct constituents of propolis have been identified globally through various separation and purification techniques [12]. Algeria, with its remarkable geographic diversity, rich flora, and varied environmental conditions [13], presents a unique and compelling opportunity to investigate the variations in propolis composition specific to this region. To comprehensively analyze its metabolomic profile, researchers typically employ techniques such as gas chromatography–mass spectrometry (GC-MS) for volatile and semi-volatile compounds, as well as ultra-high-performance liquid chromatography with diode array detection and electrospray ionization mass spectrometry (UHPLC-DAD-ESI/MS) for identifying polyphenolics.

Oxidative stress is a pathological state characterized by an imbalance between the production of reactive oxygen species (ROS) or free radicals and the body’s antioxidant defense mechanisms [14]. This cellular disruption significantly contributes to numerous natural processes or diseases, including aging, cellular degeneration, cancer, immune system dysfunction, and Alzheimer’s disease [7]. Given this, propolis supplementation offers a promising strategy for mitigating oxidative stress, potentially due to its diverse bioactive compounds, which demonstrate direct and indirect antioxidant effects [8]. Consequently, assessing antioxidant capacity is crucial in evaluating the potential therapeutic benefits of propolis.

The nuclear factor erythroid 2-related factor 2 (NRF2) signaling pathway is a central regulator of cellular antioxidant defenses [15]. The Kelch-like ECH-associated protein 1 (KEAP1)–NRF2 interaction can be modulated by NRF2-derived peptides [16] and small molecules targeting the Kelch domain of KEAP1 [17], as well as by non-covalent activators that disrupt their complex [18]. While the direct antioxidant effects of propolis are well documented, its potential to modulate the KEAP1–NRF2 pathway remains less explored. Recent docking studies have investigated the activation of the NRF2 pathway by inhibiting KEAP1 activity instigated by various phytochemicals. Notably, polyphenols, such as flavonoids, coumarins, catechins, alkaloids, terpenoids, and phenolic glycosides, many of which are present in propolis, have emerged as key plant-derived, non-covalent modulators within the KEAP1-NRF2 pathway [18], providing protective benefits against cardiovascular diseases [19]. To further elucidate these mechanisms, molecular docking could be used to examine interactions between propolis compounds and KEAP1. Furthermore, advancements in computational methods, such as virtual screening, are accelerating drug discovery by enabling early assessments of absorption, distribution, metabolism, excretion, and toxicity (ADME-Tox) properties [20], highlighting the importance of in silico approaches for evaluating the safety profiles of new chemical entities [21].

Beyond its antioxidant capacity, propolis also exhibits notable antibacterial activity, largely attributed to its rich phytochemical profile. This is particularly relevant given the rise of multidrug-resistant (MDR) bacteria [22]. Propolis has shown effectiveness against varied pathogenic strains, highlighting its potential as a natural alternative to conventional antibiotics [22,23].

Despite Algeria’s rich ecological diversity, the biological potential of its propolis resources remains underexplored. In light of the growing demand for natural alternatives in drug discovery, this study investigates propolis source from two distinct climatic regions (one arid and one semi-arid region). We characterized their chemical composition, assessed antioxidant activity, performed in silico docking analysis, and evaluated antibacterial effects against reference strains. Additionally, ADME-Tox predictions identified safe, bioactive candidates with potential for therapeutic use.

## 2. Materials and Methods

### 2.1. Chemicals and Reagents

All chemicals and reagents used were of analytical or HPLC grade. Acetonitrile and formic acid were acquired from Panreac (Barcelona, Spain). Dichloromethane (DCM) and methanol were obtained from VWR (Radnor, PA, USA). Pyridine (≥99%), trimethylchlorosilane (≥99%), N,O-bis(trimethylsilyl) trifluoroacetamide (BSTFA, ≥99%), 1,1-diphenyl-2-picrylhydrazyl (DPPH), 2,2′-azinobis(3-ethylbenzothiazoline-6-sulfonic acid) (ABTS), trichloroacetic acid (TCA), iron(III) chloride (FeCl_3_), 1,10-phenanthroline, potassium ferricyanide (K_3_Fe(CN)_6_), butylated hydroxyanisole (BHA), butylated hydroxytoluene (BHT), ascorbic acid, and quercetin were purchased from Sigma-Aldrich (St. Louis, MO, USA). The Folin–Ciocalteu reagent was procured from Biochem Chemopharma (Cosne-Cours-sur-Loire, France). All phenolic standards used for identification and quantification were supplied by Extrasynthèse (Genay, France). Ultrapure water was produced using a Direct-Q^®^ water purification system (Merck Life Science, Darmstadt, Germany).

### 2.2. Propolis Samples

Two propolis samples sourced from A. mellifera bee hives were collected by apiarists. Propolis 1 (P1) and Propolis 2 (P2) were sourced from the provinces of Batna and Biskra, respectively, during late June to late July 2020. The approximate geographical coordinates, altitude, and climatic data for each collection site are provided in Supplementary Table S1, and the sampling locations are shown on the geolocalization map depicted in Figure S1. For each location, propolis was carefully scraped from flexible grids placed at the top of multiple hives and then pooled to form a single composite sample. In the laboratory, manual cleaning was carried out to remove all impurities. Then, 100 g quantities of both propolis samples were stored in airtight containers at 4 °C until analysis.

### 2.3. Dry Propolis Extract

The ethanolic extracts of P1 and P2 were prepared with slight modifications to the procedure described by Jug et al. [24]. In brief, 100 g of each crude propolis sample was subjected to three sequential macerations, each for 3 days, using 70% ethanol at a 1:10 (*w*/*v*) ratio. Extractions were performed at room temperature in the dark, with intermittent shaking. Then, the obtained ethanolic mixtures were filtered and concentrated in a rotary evaporator. The resulting crude extracts, designated as propolis extract 1 (PE1) and propolis extract 2 (PE2), were subsequently lyophilized using a freeze-dryer (Martin Christ Alpha 2-4 LSCplus, Osterode am Harz, Germany) for complete solvent removal and long-term conservation.

### 2.4. Determination of the Total Phenolic Content (TPC) and the Total Flavonoid Content (TFC)

TPC was determined via a modified Folin–Ciocalteu method [25]. First, 20 μL of dry propolis extract was mixed with 100 μL Folin–Ciocalteu reagent (1:10) and 75 μL 7.5% of sodium carbonate (Na_2_CO_3_), before being incubated for 2 h. Absorbance was read at 765 nm using a microplate reader (Thermo Scientific™ Multiskan™ Sky, Waltham, MA, USA) with SkanIt™ Upgrade package (version 5.0). Quantification used a gallic acid calibration curve (y = 0.005x + 0.09, R2 = 0.995), with results expressed as a milligram of gallic acid equivalents per gram of dry extract (GAEs mg g^−1^). Assays were performed in triplicate.

TFC was measured using the method devised by Moreno et al. [26]. First, 50 μL of propolis extract was mixed with 50 μL of aluminum trichloride (μL AlCl_3_) and 150 μL of sodium acetate (CH_3_COONa), followed by 2.30 h of incubation in the dark. Absorbance was measured at 440 nm. A quercetin calibration curve (y = 0.0048x + 0.0121, R2 = 0.997) was used for quantification, with results expressed as milligrams of quercetin equivalents per gram of dry extract (QEs mg^−1^). Assays were performed in triplicate.

### 2.5. Advanced Analytical Techniques

#### 2.5.1. GC-MS

Propolis extracts were silylated following Oliveira et al. [27]. In brief, 20 mg of each sample was derivatized via dissolution in 450 µL of dichloromethane, adding 500 µL of docosane as an internal standard (IS), 250 µL of pyridine, 250 µL of N, O-bis(trimethylsilyl) trifluoroacetamide (BSTFA), and 50 µL of trimethylchlorosilane (TMSCl). The mixture was incubated at 70 °C for 30 min, cooled, and then analyzed via GC-MS (triplicate injections). The GC–MS analyses were performed on a Shimadzu GCMS-QP2010 Ultra (Shimadzu Corporation, Kyoto, Japan) equipped with a ZB-5ms capillary column (30 m × 0.25 mm, 0.25 μm film thickness). Helium was used as the carrier gas at a flow rate of 1.17 mL/min. The injector temperature was 320 °C, and a split ratio of 10:1 was used for injections. The oven temperature program was as follows: an initial temperature of 90 °C was held for 4 min, then increased at 16 °C/min to 180 °C, at 6 °C/min to 250 °C, and, finally, at 3 °C/min to 300 °C; the latter temperature was held for 5 min. The total run time was 42.96 min. The transfer line temperature was set to 200 °C. For mass spectrometry, Electron Ionization (EI) was used at 70 eV, with a scan range extending from *m*/*z* 50 to 1000 (1 scan/s). Compounds were identified using the NIST14 and WILEY mass spectral libraries.

#### 2.5.2. UHPLC-DAD-ESI/MS

Propolis extracts were analyzed using an Ultimate 3000 UHPLC system (Dionex Co., San Jose, CA, USA) coupled to an LTQ XL mass spectrometer (Thermo Scientific, San Jose, CA, USA). Separation was performed on a Hypersil Gold C18 column (100 × 2.1 mm, 1.9 μm). The mobile phase consisted of (A) 0.1% (*v*/*v*) formic acid in water and (B) acetonitrile. The flow rate was 0.2 mL/min and the injection volume was 10 μL (1 mg of extract/mL ethanol). Mass spectrometry analysis was performed using electrospray ionization (ESI) with a mass scan range of *m*/*z* 100–2000. The nebulizing gas used was nitrogen (>99% purity, 520 kPa), and helium was used as the collision gas. The collision-induced dissociation (CID) energy was set to 25–35 arbitrary units. Compounds were identified by matching retention times and MS/MS spectra to standards and the literature data. Quantitation was performed using an external standard method based on MS^2^ calibration curves.

### 2.6. Antioxidant Activity Evaluation

#### 2.6.1. 2,2-Diphenyl-1-picrylhydrazyl (DPPH) and 2,2′-Azino-bis (3-Ethylbenzothiazoline-6-sulfonic Acid) (ABTS) Radical Scavenging Assays

The DPPH radical scavenging activity of the extracts was evaluated using the method described by Kurek-Górecka et al. [28]. In a 96-well microplate, 40 µL of propolis extract or standard (final concentrations: 4–6.25 × 10^−2^ mg/mL for extracts; 4–4.88 × 10^−4^ mg/mL for standards; these concentrations were achieved via two-fold serial dilutions) was combined with 160 µL of DPPH solution. After incubation in the dark for 2 h, absorbance was measured at 517 nm using a PerkinElmer EnSpire Multimode Plate reader (Waltham, MA, USA).

The ABTS radical cation decolorization assay, assessing the ability to scavenge free radicals and inhibit oxidation [29], followed the procedure outlined by Xiao et al. [30]. In brief, 40 µL of propolis extract or standard was added to 160 µL of ABTS solution in a 96-well microplate. After a 10 min period of incubation in the dark, the decolorization of the ABTS radical cation was determined by measuring absorbance at 734 nm with a spectrophotometer.

The results are expressed as the half-maximal inhibitory concentration (IC_50_) in µg/mL.

#### 2.6.2. Ferric Reducing Antioxidant Power (FRAP) and 1,10-Phenanthroline (Phen) Assays

The FRAP assay was performed according to the method described by Oyaizu [31]. In brief, a reaction mixture containing 10 µL of propolis extract, 40 µL of phosphate buffer (pH 6.6), and 50 µL of 1% potassium ferricyanide (K_3_[Fe(CN)_6_]) was incubated at 50 °C for 20 min. Following incubation, 50 µL of 10% trichloroacetic acid (TCA) and 40 µL of water were added, followed by 10 µL of 0.1% iron (III) chloride (FeCl_3_). After mixing, absorbance was determined at 700 nm.

The Phen assay was performed as described by Szydłowska-Czerniak et al. [32]. First, 10 µL of propolis extract was mixed with 50 µL of FeCl_3_ (0.2%) and 30 µL of phenanthroline (0.5%), followed by the addition of 110 µL of methanol. The mixture was incubated in the dark for 20 min at 30 °C. Absorbance was then determined at 510 nm. The results are presented as A_0.50_, representing the concentration producing an absorbance of 0.500.

### 2.7. Antibacterial Activity Assessment

The minimum inhibitory concentration (MIC) and minimum bactericidal concentration (MBC) of propolis samples were evaluated against *Staphylococcus aureus* (*S. aureus*) ATCC 25923 and *Escherichia coli* (*E. coli*) ATCC 25922 via the agar dilution method, as described by Akinpelu et al. [33]. The MIC values were defined as the lowest propolis concentration (300 to 9 µg/mL) preventing visible bacterial growth on Mueller–Hinton agar plates after 24 h at 37 °C.

For MBC, samples from non-growth MIC plates were sub-cultured onto fresh nutrient agar. The MBC was defined as the lowest extract concentration that killed ≥99.9% of the initial bacterial inoculum, determined by the absence of growth on solid media after 72 h of incubation at 37 °C.

### 2.8. Computational Analysis

#### 2.8.1. Data Insights

Statistical analyses were performed using IBM SPSS Statistics 29 and XLSTAT 2016, and R software (version 4.0.0). The results are presented as the mean ± standard deviation (SD) from three independent experiments, each conducted in triplicate. IC_50_ values were determined via linear regression. A one-way analysis of variance (ANOVA) was used to evaluate the effect of geographic origin on antioxidant activity and to compare propolis extracts with reference standards. Tukey’s HSD post hoc test was applied to identify significant pairwise differences (*p* < 0.05).

Principal component analysis (PCA) was conducted on standardized data to explore sample distribution and highlight influential variables. Biplots of PC1 and PC2 were used to visualize sample clustering and variable contributions.

#### 2.8.2. In Silico Molecular Modeling

The X-ray crystal structure of the KEAP1 Kelch domain (PDB ID: 4L7B), complexed with ML334 [(1S,2R)-2-[(1S)-1-[(1,3-dioxoisoindol-2-yl)methyl]-3,4-dihydro-1H-isoquinoline-2-carbonyl]cyclohexane-1-carboxylic acid)], was selected for molecular docking studies due to its good resolution (2.41 Å) and co-crystallization with a reference ligand, which provides an optimal framework for validating our docking protocol. The structure was retrieved from the Protein Data Bank (https://www.rcsb.org/ (accessed on 24 March 2024)). This structure was prepared using the Protein Preparation Wizard in Maestro (v11.8) with the OPLS3e force field, as per Bourougaa et al. [34]. Potential ionization states and partial atomic charges were set at pH 7.0. Geometry was refined via restrained minimization to a Root Mean Square Deviation (RMSD) of 0.3 Å, consistent with Keighobadi et al. [35]. Additional validation was performed using the Dock RMSD online tool (https://zhanggroup.org/DockRMSD/ (accessed on 1 April 2024)). This prepared structure then served as the receptor for grid generation, utilizing standard box dimensions for XP-docking.

Initial 2D structures of the 28 bioactive components were retrieved from the PubChem database (https://pubchem.ncbi.nlm.nih.gov/, (accessed on 7 June 2025)) and ChemDraw 23.1 and saved as PDB files. Their 3D structures were constructed and energy-minimized using the LigPrep module of Schrödinger Suite (v11.8).

Finally, the prepared molecules (ML334 and the 28 bioactive components) were docked into the active site of KEAP1 (PDB ID: 4L7B, https://www.rcsb.org/structure/4L7B (accessed on 24 March 2024)) using the extra-precision (XP-docking) feature of Maestro’s Glide module. Interactions were visualized with Discovery Studio Visualizer to illustrate the interactions between the bioactive compounds and the active site of KEAP1 to assess the inhibitory activity of the Algerian propolis extract molecules, as described by Bourougaa et al. [34].

#### 2.8.3. In Silico ADME-Tox Predictions

The ADME properties of each component were predicted using the SwissADME web tool (http://www.swissadme.ch, (accessed between 10 and 20 April 2024)) [36], focusing on solubility and membrane permeability, key factors influencing bioactivity [34]. The SMILES (Simplified Molecular Input Line Entry System) notation of the chain core, obtained from PubChem (https://pubchem.ncbi.nlm.nih.gov/, (accessed on 9 April 2024)), was used as input for the SwissADME predictions. Toxicological profiles, including acute toxicity, hepatotoxicity, cytotoxicity, immunotoxicity, and LD_50_ values, were assessed using the ProTox-II (https://tox-new.charite.de/protox_II/), (accessed between 15 and 20 April 2024)) [21].

## 3. Results

### 3.1. TPC and TFC

The Algerian propolis extracts showed significant differences in phenolic and flavonoid contents. The Batna sample (PE1) exhibited higher concentrations of both total phenolics (317.87 ± 1.85 mg GAE/g) and flavonoids (50.68 ± 4.09 mg QE/g) compared to the Biskra sample (PE2; 238.05 ± 1.93 mg GAE/g and 43.47 ± 0.96 mg QE/g, respectively; *p* < 0.05). All values, expressed as gallic acid equivalents (GAEs) and quercetin equivalents (QEs) per gram of dry extract, represent the means of triplicate measurements with statistically significant variation (Tukey test).

### 3.2. Chromatographic Analysis Results

#### 3.2.1. GC-MS Results

The total ion current (TIC) chromatogram is illustrated in Figure S2, and identified chemical constituents are systematically detailed in Table 1. PE1 comprised 34 distinct compounds, with relative abundances ordered as follows: terpenoids > carboxylic acids > sugars > fatty acids > steroids and alcohols. In contrast, PE2 contained 36 different compounds, showing relative abundances ordered as follows: terpenoids > fatty acids > sugars > carboxylic acids > alcohols and steroids. Both extracts shared 13 common compounds with varying quantities. Pimaric acid (a diterpene) was the most abundant compound in both extracts.

#### 3.2.2. UHPLC-DAD-ESI/MS Results

Chromatograms illustrating the results are presented in Figure S3; the extract compositions are detailed in Table 2. PE1 contained 20 phenolic compounds, while PE2 had 48 phenolic compounds. Notably, 14 phenolic compounds were common to both extracts, though their concentrations varied. Compounds were categorized as phenolic acids and flavonoids.

The most abundant compounds in both extracts included caffeic acid isoprenyl I, pinobanksin-3-*O*-acetate, caffeic acid isoprenyl III, pinobanksin, and caffeic acid isoprenyl II. However, their relative abundances and concentrations differed notably between the two extracts.

### 3.3. Antioxidant Activity

The antioxidant activity of propolis extracts was assessed using radical scavenging (DPPH, ABTS) and reducing power (FRAP, phenanthroline) assays. The results are expressed as the half-maximal inhibitory concentration (IC_50_) and the concentration producing an absorbance of 0.500 (A_0.5_), which quantify the efficiency of an extract in neutralizing free radicals or reducing oxidants. Lower IC_50_ or A_0.5_ values indicate stronger antioxidant activity.

**Figure 1:**
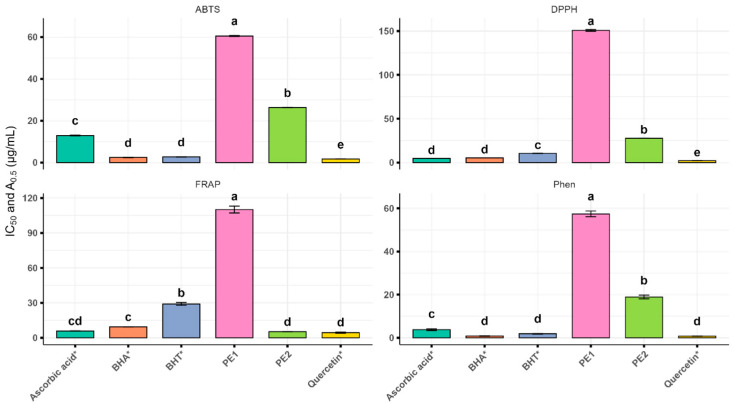
IC_50_ values (DPPH, ABTS) and A_0.5_ values (FRAP, Phen) of Algerian propolis extract. The values with different superscripts (a, b, c, d, or e) in the same columns are significantly different (*p* < 0.0001) based on analysis of variance, followed by Tukey’s honestly significant difference test. The samples marked with an asterisk (*) are the reference compounds (standards).

As shown in Table 3 and Figure 1, PE1 and PE2 exhibited significantly different antioxidant capacities across all assays (*p* < 0.0001). In the DPPH assay, PE2 was approximately five times more potent than PE1, while in the ABTS assay, its activity was nearly two times higher. The reducing power assays confirmed this trend: in FRAP, PE2 showed a 21-fold higher activity than PE1, exceeding the activities of the reference standards butylated hydroxyanisole (BHA) and butylated hydroxytoluene (BHT) and approaching those of quercetin and ascorbic acid. The phenanthroline (Phen) assay yielded similar results, with PE2 displaying significantly greater reducing power than PE1.

### 3.4. Antibacterial Activity

The antibacterial activities of PE1 and PE2 are summarized in Table 4. Both samples demonstrated inhibitory and bactericidal effects against *S. aureus* ATCC 25923 and *E. coli* ATCC 25922. Notably, the MIC and MBC values were identical for each sample and strain, strongly suggesting a bactericidal effect. For *S. aureus*, MIC/MBC values ranged from 18.75 µg/mL (PE2) to 37.5 µg/mL (PE1), while for *E. coli*, the values ranged from 133 µg/mL (PE2) to 300 µg/mL (PE1).

### 3.5. Results of Computational Studies

#### 3.5.1. Molecular Modeling

**Figure 2:**
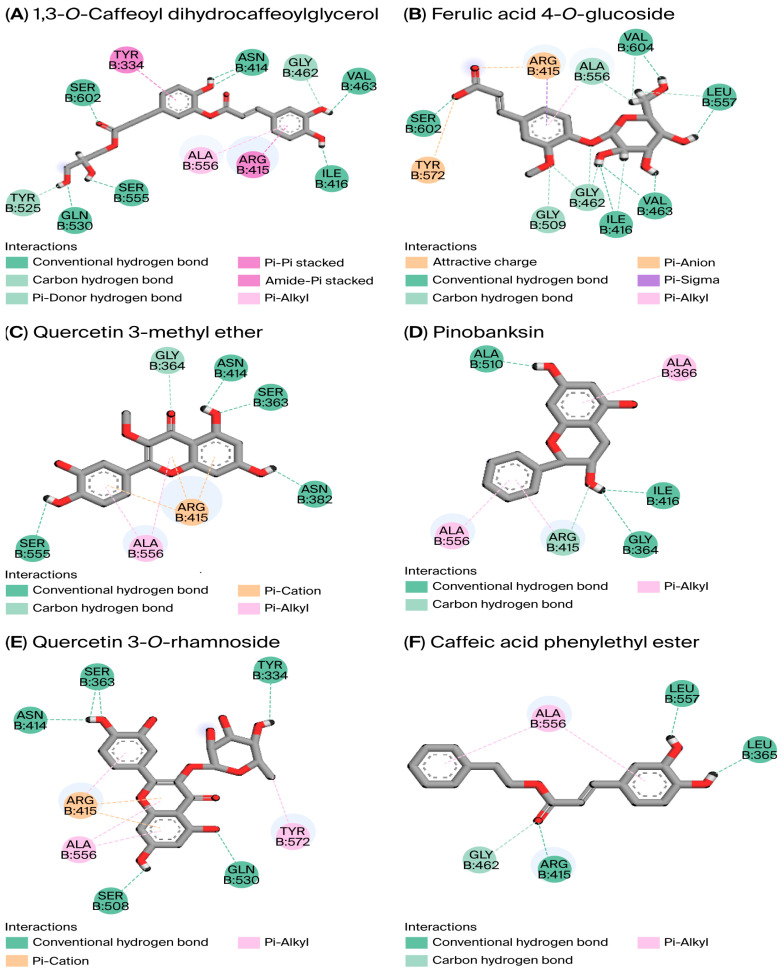

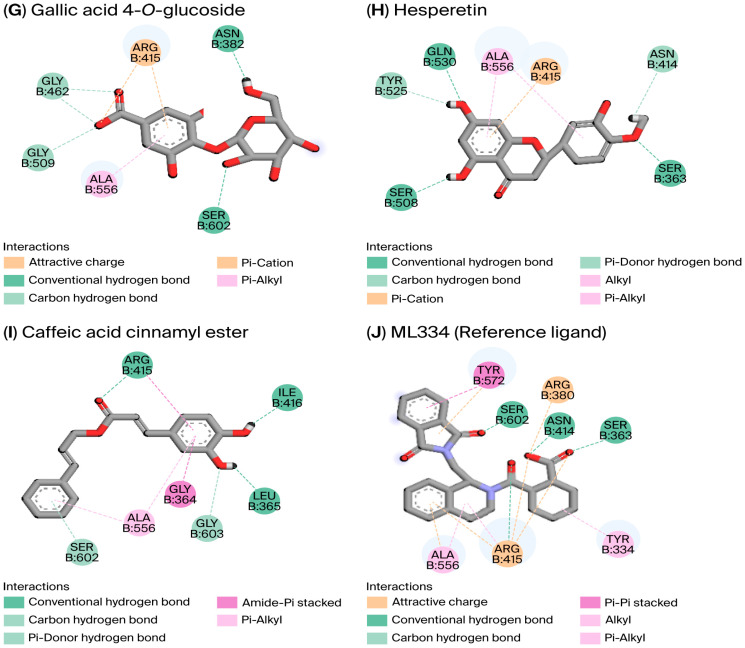
Two-dimensional diagrams showing the bioactive components of Algerian propolis extract (**A**–**I**) and ML334 (**J**) within Keap1 (PDB ID: 4L7B). Residues: Ala, alanine; Arg, arginine; Asn, asparagine; Gln, glutamine; Gly, glycine; Ile, isoleucine; Leu, leucine; Ser, serine; Tyr, tyrosine; Val, valine.

Our findings revealed that 9 of the 28 compounds assessed exhibited strong affinity for the KEAP1 binding site, disrupting the KEAP1–NRF2 complex more effectively than the reference molecule ML334 (Figure 2). Binding affinity, which quantifies interaction strength, is characterized by lower binding energy, indicating stronger interactions [37]. Our detailed binding affinity results are presented in Table 5. To validate the protein–ligand docking (PLD) outcomes, we assessed the RMSD. This common measure evaluates prediction accuracy by comparing the predicted ligand conformation with the crystallographic structure [38]. Our calculated RMSD of 0.683 Å is a good value to attain when performing virtual screening.

The calculated binding energy ranged from −11.020 to −6.971 kcal/mol, surpassing the ML334 reference molecule’s binding affinity of −6.807 kcal/mol. ML334 forms hydrogen bonds and hydrophobic interactions with the key amino acid residues Ser363, Asn414, Arg415, Ser602, Arg380, Ala556, and Tyr334. These interactions also contribute to the stability of the complexes formed between the nine bioactive compounds and the KEAP1 binding cavity. Of the top three complexes presented in Table 5, 1,3-*O*-caffeoyl-dihydrocaffeoylglycerol showed the strongest affinity for KEAP1 (−11.020 kcal/mol). It forms hydrogen bonds and hydrophobic interactions with the P1, P2, P3, P4, and P5 sub-pockets of KEAP1.

#### 3.5.2. In Silico ADME-Tox Properties

The ADME-Tox properties of bioactive compounds from both Algerian propolis extracts, evaluated via molecular docking, are detailed in Table S2. These compounds, with molecular weights ranging from 272.25 to 355.32 g/mol, show high potential for effective intestinal absorption. Their log *p* values, ranging between −0.89 and 1.84, indicate optimal lipophilicity for enhanced membrane permeability. Aqueous solubility (log S) from −3.89 to −1.14 suggests adequate systemic distribution. Crucially, these compounds do not inhibit major cytochrome P450 enzymes, minimizing the risk of drug–drug interactions. Oral bioavailability scores fall within an acceptable range of 0.55–0.56, and synthetic feasibility is favorable, ranging from 2.62 to 4.46.

Regarding toxicity, predicted LD_50_ values of 435 to 5000 mg/kg place the samples within toxicity classes 4 to 5 (on a scale of 6, where class 6 is the safest).

## 4. Discussion

This research presents a comprehensive phytochemical analysis of Algerian propolis samples sourced from two climatically distinct geographic regions, Batna (humid subtropical) and Biskra (hot desert), with a particular focus on the previously unexplored area of Biskra. The findings underscore the significant impact of geography on the composition and bioactivity of phytochemicals. We used a combination of analytical, biological, and computational modeling to determine the extracts’ antioxidant and antibacterial capabilities, as well as their molecular interactions with the KEAP1-NRF2 signaling pathway.

Our analysis revealed significant variability in TPC and TFC, with PE1 (Batna) exhibiting higher concentrations than PE2 (Biskra). These TPC values are comparable to those of other Algerian propolis and Turkish/Brazilian brown propolis, though lower than those of the Brazilian green and red varieties [39,40,41]. The TFC values are considerably higher than some previously reported for Algerian propolis [39,42] but lower than those documented by Ouahab et al. [43] and for Brazilian green/red propolis [41]. The observed differences between PE1 and PE2 could suggest a potential influence of geographic and climatic variations on propolis composition, possibly through their impact on local flora. The relatively high TPCs and TFCs of our extracts highlight their promising commercial potential, given their established link to various biological activities, especially antioxidant properties [44]. These findings need to be confirmed in a future study involving a larger sample size in these two regions.

GC-MS analysis identified 34 distinct compounds in PE1 and 36 distinct compounds in PE2 (Table 1), with 13 common components. Predominant compounds include diterpenes (pimaric acid, isopimaric acid, 7-β-hydroxydehydroabietate methyl), caffeic acid (carboxylic acid), butanedioic acid (fatty acid), naringenin (flavanone), various sugars, androsta-3,5-dien-3,17-dione (steroid), and glycerol (alcohol). The geographic origin seems to significantly shape the chemical composition of these propolis samples, leading to variations in biological activities. Our analysis indicates that our propolis samples from Biskra and Batna are predominantly rich in diterpenes, consistent with other North African and Eastern Mediterranean propolis [3]. These diterpenes, notably pimaric acid (the most abundant constituent in both PE1 and PE2), are recognized for their diverse properties, including antibacterial, antiviral, cytotoxic, anticancer, and anti-inflammatory effects [45,46]. The identified fatty acids also contribute to propolis’s nutritional value and antioxidant effects [47]. While our findings show similarities to previous Algerian investigations, significant variations in other studies [48] underscore Algeria’s rich natural heritage and biodiversity. Furthermore, Algerian propolis shares compositional similarities, particularly in its diterpene dominance, with propolis from diverse global regions such as Turkey [49] and brown propolis from southern Brazil [50]. This diterpene-rich profile is characteristic of propolis types originating from various tropical and Mediterranean countries [3,51].

The UHPLC-DAD-ESI/MS analysis further elucidated the phenolic profile of our propolis extracts (Table 2). We identified 16 compounds, including various phenolic acids (caffeic, *p*-coumaric, ferul), caffeic acid esters (isoprenyl, phenylethyl, cinnamyl), and flavonoids (pinobanksin, kaempferol, kaempferide, apigenin, quercetin, chrysin, hesperetin, isorhamnetin), along with pinobanksin acetates and propionates. These compounds are commonly reported in propolis originating from Algeria [9,52], Turkey [53], Brazil [54], and Ecuador [55]. PE1 and PE2 shared 14 compounds, with caffeic isoprenyl ester, pinobanksin, and pinobanksin-3-*O*-acetate being the most abundant. Additionally, we detected several phenolic and flavonoid glycosides, compounds rarely reported in propolis prior to 2011, with occurrences reported mainly in Brazil, Serbia, Portugal, and the United Kingdom [3].

This study marks the first identification of two phenylpropanoids in Algerian propolis (PE2). The first, *p*-methoxycinnamic acid cinnamyl ester, has previously been reported in propolis originating from Australia, Brazil, Egypt, and Uruguay [3]. The second, 1,3-*O*-caffeoyl dihydrocaffeoylglycerol (a phenylpropanoid glyceride), is reported for the first time in propolis in this manuscript. This novel compound, a hydroxycinnamic acid derivative, has previously been found in sorghum grain [56] and the Mediterranean shrub Myrtus communis [57]. We hypothesize that its presence in PE2 is linked to the sorghum plant, which is native to Africa and well adapted to the arid environmental conditions of the Biskra region from which PE2 was sourced [56].

Our UHPLC-DAD-ESI/MS analysis revealed pronounced variability between the two propolis extracts. PE2 exhibited significantly higher concentrations and a qualitatively richer profile of phenolic acids and flavonoids compared to PE1. These differences, also observed in our GC-MS analysis, could be largely attributable to substantial variations in geographic regions, botanical sources, and the distinct local abiotic and biotic factors that impact the biosynthetic pathways of the plants foraged by bees [6]. Despite concurrent seasonal collection, these environmental differences likely contributed to the observed compositional variations. These findings need to be confirmed in a larger study with a larger sample size and multiple sampling periods across all seasons.

Besides the direct geographic and botanical influences, altitude appears to be a significant factor shaping propolis composition. Sorucu and Oruç [58] proposed that lower altitudes generally favor the accumulation of certain secondary metabolites. Supporting this, Anđelković et al. [59] observed a greater prevalence of flavonoids at lower altitudes, while phenolic glycerides were more abundant at higher altitudes. Neto et al. [60] further highlighted altitude as a critical geographic factor influencing these variations. Given these insights, we hypothesize that the lower altitude of PE2 (121 m) contributes to its greater richness in secondary metabolites compared to PE1 (1045 m).

Furthermore, climatic conditions and their fluctuations have a crucial influence on propolis composition. Neto et al. [60] noted that humid phases favor primary metabolites, while drier periods lead to elevated levels of secondary metabolites in plants. Similarly, regional climate changes, particularly rainfall and drought episodes, have a significant relationship with flavonoid concentrations. Thus, variations in environmental conditions such as water availability, humidity, solar radiation, insect interactions (bees), and pathogen pressures can drive plants to respond with altered physiological characteristics and secondary metabolite production [6]. These complex environmental dynamics should be considered in future efforts to standardize propolis products.

As Hossain et al. [10] emphasized for reliable assessment, we utilized multiple antioxidant assays, each operating through distinct mechanisms (DPPH, ABTS, FRAP, and Phen). Significantly different activity (*p* < 0.0001) was observed between PE1 and PE2, with PE2 being consistently stronger overall. Specifically, PE2 demonstrated high activity across all assays, while PE1 showed robust ABTS/Phen but moderate DPPH/FRAP activity. These findings corroborate our characterization analyses, reflecting considerable qualitative and quantitative phytochemical differences.

Compared to prior studies on Algerian propolis, our extracts exhibited higher antioxidant activities than samples from other locations [39], yet these activity levels were lower than those from northeast Algeria [43]. Notably, PE2’s Phen assay results aligned closely with Hadjab et al. [61], while its DPPH activity mirrored Ayad et al. [52]. However, FRAP values in our study exceeded those reported by Teggar et al. [42]. Collectively, these findings underscore the variability in Algerian propolis’s antioxidant properties, driven by region-specific chemical compositions shaped by geographic and floristic factors.

Regarding antibacterial assessment, both PE1 and PE2 exhibited notable inhibitory activity against *S. aureus* and *E. coli*. A key finding was the identical MIC and MBC values, strongly suggesting a direct bactericidal effect. This highlights our propolis samples’ potent capacity to kill bacterial cells at relatively low concentrations. PE2 consistently demonstrated stronger activity than PE1 against both strains. Our findings align with some Algerian studies on *S. aureus* [62] and demonstrate higher activity than identified in other studies [9]. They are also comparable with the results reported by Segueni et al. [40] for both bacterial strains, confirming the high general activity of Middle Eastern propolis compared to other types originating from other global regions [40,63]. Consistent with broader propolis research, activity was significantly higher against the Gram-positive *S. aureus* than the Gram-negative *E. coli*, primarily due to cell wall structural differences [63]. This promising efficacy suggests that our Algerian propolis samples may have significant potential as natural antimicrobial agents.

We performed PCA on UHPLC-DAD-ESI/MS-identified bioactive compounds to understand their relationship with antioxidant properties (Figure S4). The first two principal components (PC1 and PC2) collectively explained 87.2% of the total variance. Component 1 (PC1) alone accounted for 66.9% of the total variance and had a strong correlation with antioxidant activity (negative contributions from DPPH, ABTS, FRAP, and Phen).

In the PCA score plot, samples segregated distinctly along PC1 based on their antioxidant potential: PE2 samples (higher activity) clustered on the positive PC1 side, while PE1 samples (lower activity) clustered on the negative PC1 side. The loading plot revealed that the positive PC1 side (right) was enriched with phenolic compounds predominantly associated with PE2, including *p*-coumaric acid, caffeic acid cinnamyl ester, *p*-methoxycinnamic acid cinnamyl ester, and chrysin. Conversely, the negative PC1 side (left) was characterized by PE1-specific compounds such as quercetin, a caffeic acid derivative, and elevated kaempferol levels.

Notably, TPC and TFC showed a negative correlation with the antioxidant activity axis (PC1), contrasting with much of the literature but aligning with reports by Laaroussi et al. and Wang et al. [44,64]. This suggests that TPC/TFC alone may not predict antioxidant efficacy, likely due to complex interactions among propolis compounds (e.g., synergism or antagonism) [65].

PC2 (20.2% variance) further differentiated samples: its positive side correlated with kaempferide and pinobanksin-3-*O*-propionate, while the negative side correlated with quercetin-3-methyl ether. This highlights region-specific compositional differences in propolis, with PC1 reflecting antioxidant capacity and PC2 reflecting distinct biochemical markers.

We employed an in silico approach to investigate interactions between UHPLC-DAD-ESI/MS-identified bioactive compounds (PE1/PE2) and the KEAP1-NRF2 pathway (PDB:4L7B). Key compounds, including quercetin derivatives and caffeic acid esters, formed hydrogen bonds and hydrophobic interactions with KEAP1’s P1-P5 sub-pockets, engaging critical residues (Tyr334, Arg415, and Ser508) [16,17]. Notably, PE2, which showed higher antioxidant activity in vitro, contained multiple high-affinity binders (e.g., 1,3-*O*-Caffeoyl-dihydrocaffeoylglycerol, quercetin-3-methyl-ether, quercetin-3-*O*-rhamnoside, caffeic acid cinnamyl ester and gallic acid 4-*O*-glucoside), suggesting a mechanistic basis for its enhanced bioactivity. These interactions mirrored those of established NRF2 modulators (e.g., apigenin binding Arg415/Tyr334/Ser602 [18]), suggesting the potential for shared activation mechanisms. Given the critical role of these residues in KEAP1-NRF2 binding, our model predicts that targeted disruption of the complex by constituents of PE2 might efficiently promote NRF2-mediated antioxidant responses. Our findings align with current pharmacological strategies against oxidative stress disorders [15,18,66] and underscore PE2’s therapeutic potential due to its residue-specific binding profile.

ADME-Tox profiling is critical for addressing key drug discovery challenges, including efficacy, safety, and cost attrition [36]. Many candidates fail in preclinical development due to poor pharmacokinetics, toxicity, or insufficient target engagement, leading to significant financial and time losses [20]. Our analysis (Table S2) demonstrates that all evaluated compounds except quercetin 3-*O*-rhamnoside comply with Lipinski’s rule of five [67], indicating promising drug-likeness and potential oral bioavailability for further development.

Finally, as a preliminary study, these findings should be interpreted with consideration of certain limitations, including the small sample size, seasonal variations, and environmental factors that may influence propolis composition and bioactivity, potentially leading to inconsistencies across seasons or regions. Nevertheless, these results provide useful insights, underscoring the need for future large-scale studies with standardized collection protocols, broader geographical sampling, and clinical validation to fully assess Algerian propolis’s therapeutic potential.

## 5. Conclusions

This study on propolis from Batna and Biskra, Algeria, revealed distinct phytochemical profiles linked to geographic origin, along with potent antioxidant and bactericidal effects against *S. aureus* and *E. coli*. Notably, phenylpropanoids, including 1,3-*O*-caffeoyl dihydrocaffeoylglycerol, a compound newly identified in propolis, were detected in PE2, a sample sourced from a previously unexplored region. In silico molecular docking suggested the potential of its high-affinity constituents as promising modulators of the KEAP1-NRF2 pathway, with a favorable ADME-Tox profile. This research highlights Algerian propolis as a valuable natural product source of diverse bioactive compounds. By identifying a novel compound and providing predictive mechanistic insights, our findings lay a solid foundation for drug discovery and further therapeutic investigations into oxidative stress and antimicrobial resistance.

## Figures and Tables

**Table 1:** The volatile compounds present in the Algerian propolis extracts detected via gas chromatography–mass spectrometry (GC-MS).

Peak	Rt (min)	Profiled Volatile	Quantification (µg Compound/g Extract)		*p*-Value
			PE1	PE2	
1	6.74	Verbenyl ethyl ether	29.99 ± 0.01	-	-
5	8.22	α-Pinene	12.81 ± 0.03	-	-
7	8.44	α-Terpineol	55.75 ± 0.01	-	-
8	8.72	α-Terpinyl acetate	14.65 ± 0.01	-	-
11	10.38	δ-Cadinene	12.35 ± 0.01	-	-
12	10.74	β-Terpineol	35.01 ± 0.05	-	-
13	10.80	Cubebol	14.64 ± 0.01	-	-
14	11.51	Valerianol (4b*H*,5a-Eremophil-1(10)-ene)	28.88 ± 0.01	-	-
30	15.98	(13*R*)-8,13-Epoxylabd-14-ene	17.78 ± 0.04	-	-
35	17.34	Pimaric acid	1631.19 ± 0.07	1505.00 ± 0.65	0.756
39	20.36	Isopimaric acid	404.72 ± 0.01	298.50 ± 0.16	0.319
45	21.00	Callitrisic acid	98.39 ± 0.02	-	-
42	20.71	4-epi-Abietic acid	22.13 ± 0.07	-	-
43	20.97	Dehydroabietic acid	-	95.27 ± 0.03	-
47	21.93	Methyl 7-β-hydroxydehydroabietate	116.48 ± 0.09	41.96 ± 0.01	0.001
48	22.38	Farnesol	-	9.08 ± 0.02	-
55	36.56	α-Amyrin	-	24.87 ± 0.01	-
56	38.15	Methyl commate C	-	12.82 ± 0.04	-
57	38.85	Lupeol	-	84.97 ± 0.04	-
**Total terpenoids**			2494.77 μg/g	2072.47 μg/g	0.383
4	8.04	Dihydrocinnamic acid	26.68 ± 0.01	-	-
10	9.93	Malic acid	-	148.60 ± 0.06	-
20	13.38	Citric acid	-	118.51 ± 0.01	-
22	13.85	Quininic acid	-	74.02 ± 0.03	-
26	15.01	10,12-Docosadiynedioic acid	344.62 ± 0.11	-	-
31	16.10	3,4-Dimethoxycinnamic acid	-	39.01 ± 0.01	-
33	16.79	Ferulic acid	-	55.10 ± 0.01	-
40	20.53	Caffeic acid	151.81 ± 0.03	297.22 ± 0.03	0.004
**Total carboxylic acids**			523.11 μg/g	732.44 μg/g	0.004
6	8.29	Butanedioic acid	55.75 ± 0.03	105.95 ± 0.05	0.205
19	13.50	Myristoleic acid	273.55 ± 0.07	-	-
32	16.37	Palmitic Acid	-	408.71 ± 0.14	-
36	18.596	Linoleic acid	-	436.92 ± 0.10	-
37	18.68	Oleic acid	-	994.56 ± 0.15	-
41	20.61	Oleamide	NQ	-	-
**Total fatty acids**			329.30 μg/g	1946.14 μg/g	0.205
17	12.85	D-Tagatose	-	62.95 ± 0.02	-
18	13.35	D-Fructose	90.64 ± 0.73	394.83 ± 0.15	0.032
21	13.63	D-Talose	-	43.39 ± 0.01	-
23	14.22	D-Psicose	50.26 ± 0.09	128.86 ± 0.04	0.031
24	14.28	D-Mannose	73.44 ± 0.02	102.69 ± 0.06	
25	14.83	D-Galactose	43.75 ± 0.01	49.04 ± 0.01	0.505
28	15.38	D-Glucose	93.70 ± 0.02	147.76 ± 0.05	0.769
52	24.36	Sucrose	-	96.33 ± 0.02	-
53	24.88	Methyl galactoside	-	101.45 ± 0.02	-
54	26.52	Mannobiose	-	54.98 ± 0.03	-
**Total sugars**			351.79 μg/g	1095.64 μg/g	0.045
38	20.24	Allocholic acid	38.99 ± 0.04	-	-
44	20.81	17-Methylandrosta-1,4-dien-3-one	26.59 ± 0.01	-	-
51	23.82	Androsta-3,5-diene-3,17-dione	43.16 ± 0.05	34.27 ± 0.07	0.151
**Total steroids**			108.74 μg/g	34.27 μg/g	0.151
3	7.81	Glycerol	15.26 ± 0.04	52.58 ± 0.03	0.140
27	15.09	*scyllo*-Inositol	7.43 ± 0.01	-	-
34	16.88	*myo*-Inositol	-	107.60 ± 0.04	-
**Total alcohols**			22.69 μg/g	160.18 μg/g	0.140
2	7.32	Epimethendiol	127.19 ± 0.08	-	-
9	9.61	1-Methyl 2-cyclohexene-1-methanol	5.47 ± 0.02	-	-
15	11.53	Gluconolactone	-	NQ	-
16	12.54	2-Furanacetaldehyde	-	NQ	-
29	15.66	Galactonic acid	-	86.65 ± 0.02	-
49	23.42	Retroretinol	15.78 ± 0.01	-	-
50	23.53	Naringenin	NQ	NQ	-
**Other compounds**					

The results are presented as the mean ± standard deviation. (-), not detected; NQ, not quantified; PE1, propolis extract 1; PE2, propolis extract 2; Rt, retention time.

**Table 2:** The phenolic compounds in the Algerian propolis extracts identified using ultra-high-performance liquid chromatography with diode array detection and electrospray ionization mass spectrometry (UHPLC-DAD-ESI/MS).

	Profiled Molecule	Rt (min)	λ (nm)	[M-H]^−^	Quantification (µg/g Extract)		*p*-Value
					PE1	PE2	
1	Unknown	1.66	208, 258, 282, 319	377			-
2	Caffeic acid	2.38	208, 324	179	572.08 ± 0.04	-	0.003
		2.40	208, 290, 323		-	1666.71 ± 0.44	
3	*p*-Coumaric acid	3.44	208, 312	163	-	511.68 ± 0.27	-
4	Ferulic acid derivative	4.09	208,323	397	277.47 ± 0.17	-	
		7.45	208, 324		137.57 ± 0.07	-	0.023
		7.54	208, 322			456.98 ± 0.20	
5	Gallic acid 4-*O*-glucoside	17.37	208, 311	331 (377)	-	NQ	-
6	Caffeic acid isoprenyl ester I	21.44	209, 269, 322	247	3518.39 ± 0.14	-	<0.0001
		21.54	208, 268, 320		-	12,127.35 ± 0.02	
7	Caffeic acid isoprenyl ester II	22.27	207, 299, 325	247	2572.2 ± 1.63	-	0.349
		22.37			-	6820.26 ± 2.77	
8	Caffeic acid isoprenyl ester III	22.85	208, 290, 330	247	2743.55 ± 0.22	-	0.964
		22.93			-	8932.12 ± 1.39	
9	Caffeic acid phenylethyl ester	25.39	209, 326	283	449.38 ± 0.46	-	0.157
		25.50	208, 295, 326		-	2928.35 ± 1.62	
10	*p*-Coumaric acid isoprenyl ester	30.21	208, 314	231	-	728.37 ± 0.30	-
11	Caffeic acid cinnamyl ester	30.68	207, 294, 326	295	-	842.25 ± 0.39	-
12	*p*-Coumaric acid hexose	35.60	209, 290, 328	325	-	NQ	-
13	Caffeic acid derivative	36.85	209, 263, 318	315 (361)	314.24 ± 0.14	-	-
14	Ferulic acid 4-*O*-glucoside	39.21	208, 293, 335	355	-	NQ	-
15	*p*-Methoxycinnamic acid cinnamyl ester	41.55	206, 281	293 (339)	-	282.75 ± 0.35	-
16	Benzofuran derivative	45.83	229, 278	301	1273.47 ± 0.23	-	0.064
		45.86	231, 276		-	361.13 ± 0.07	
17	Ferulic acid derivative	47.35	252, 326	517	-	584.66 ± 0.12	-
	Total phenolic acids				11,858.35 µg/g	35,448.18 µg/g	0.550
18	Querceti-3-*O*-rhamnoside	4.15	208, 297, 343	447	-	NQ	-
19	Pinobanksin-5-methyl ether	10.16	208, 287	285	-	1257.76 ± 1.28	-
20	Quercetin-3-methyl-ether	10.74	207, 265, 355	315	-	1398.68 ± 0.61	0.604
		36.17	207, 267, 353	315 (361)	1263.72 ± 0.63	-	
21	Pinobanksin	12.31	208, 292, 335	271	3140.4 ± 0.37	-	0.006
		12.41			-	7102.82 ± 0.82	
22	Kaempferol	12.80	207, 265, 368	285	1773.39 ± 1.53	-	0.598
		12.89	206, 266, 367		-	646.02 ± 0.05	
23	Kaempferide	14.27	207, 266, 351	299	292 ± 0.11	339.23 ± 0.24	0.132
24	Quercetin-dimethyl ether	19.41	207, 261, 356	329	-	470.40 ± 0.37	-
25	Apigenin	23.43	207, 266, 358	269	1894.54 ± 0.58	-	0.036
		23.53	202, 265, 358		-	4158.55 ± 1.62	
26	Pinobanksin-3-*O*-acetate	24.84	208, 293	313	4530.01 ± 1.47	-	0.038
		24.93			-	9730.09 ± 3.59	
27	Naringenin hexoside	26.13	208, 331	433	NQ	-	-
		26.24	203, 300, 331		-	NQ	-
28	Chrysin	29.35	208, 316	253	-	1151.80 ± 0.92	-
29	Pinobanksin-3-*O*-propionate	31.78	209, 292, 328	327	470.08 ± 0.09	1121.68 ± 0.13	0.026
30	Pinobanksin-3-*O*-pentanoate	35.21	288. 329	355	NQ	-	-
		35.26	208, 268, 329		-	NQ	-
31	Hesperetin	35.56	210, 289, 368	317 (363)	NQ	-	-
32	Isorhamnetin	35.85	210, 270, 372	315	907.19 ± 0.31	2767.44 ± 0.28	0.040
33	Quercetin	47.81	255, 367	301 (347)	129.67 ± 0.09	-	-
	Total flavonoids				14,401.00 µg/g	30,144.47 µg/g	0.009
34	1,3-*O*-Caffeoyl-dihydrocaffeoylglycerol	35.88	208, 260, 294, 324	417	-	NQ	-
	Other phenolic compounds						

The results are presented as the mean ± standard deviation. Peak identification was confirmed either by comparing retention times, mass spectra, and diode array detector spectra with those of standard compounds injected under identical conditions or by referencing mass spectrometric and ultraviolet–visible data previously reported in the literature. Each identified compound was quantified using external standards and calibration curves created with the closest structurally similar pure standards. (-), not detected; [M-H]^−^, pseudomolecular ion; NQ, not quantified; PE1:, propolis extract 1; PE2, propolis extract 2; Rt, retention time.

**Table 3:** The antioxidant activity of the Algerian propolis extracts.

	IC_50_ (μg/mL)		A_0.5_ (μg/mL)	
Sample	DPPH	ABTS	FRAP	Phen
PE1	150.68 ± 1.44 ^e^	60.49 ± 0.22 ^e^	110.09 ± 2.97 ^d^	57.44 ± 1.35 ^d^
PE2	27.74 ± 0.19 ^d^	26.30 ± 0.12 ^d^	5.16 ± 0.12 ^a^	18.90 ± 1.13 ^c^
Ascorbic acid *	4.70 ± 0.01 ^b^	12.92 ± 0.18 ^c^	5.47 ± 0.20 ^a^	3.74 ± 0.17 ^b^
BHT *	10.49 ± 0.07 ^c^	2.68 ± 0.08 ^b^	28.96 ± 1.87 ^c^	1.84 ± 0.15 ^a,b^
BHA *	5.41 ± 0.01 ^b^	2.42 ± 0.11 ^b^	9.33 ± 0.06 ^b^	0.79 ± 0.09 ^a^
Quercetin *	2.12 ± 0.04 ^a^	1.52 ± 0.02 ^a^	4.38 ± 0.09 ^a^	0.75 ± 0.01 ^a^

The results are expressed as the mean ± standard deviation of three measures. The samples marked with an asterisk (*) are the reference compounds (standards). Lower values indicate higher antioxidant activity. The values with different superscripts (a, b, c, d, or e) in the same columns are significantly different (*p* < 0.0001) based on analysis of variance, followed by Tukey’s honestly significant difference test. A_0.5_: the concentration producing an absorbance of 0.500, ABTS: 2,2′-azino-bis (3-ethylbenzothiazoline-6-sulfonic acid), BHA: butylated hydroxyanisole, BHT: butylated hydroxytoluene, DPPH: 2,2-diphenyl-1-picrylhydrazyl, FRAP, ferric reducing antioxidant power, IC_50_: half-maximal inhibitory concentration, PE1, propolis extract 1, PE2: propolis extract 2, Phen: 1,10-phenanthroline.

**Table 4:** The antibacterial activities of the Algerian propolis extracts.

Sample	Strain	MIC (µg/mL)	MCB (µg/mL)
PE1	*S. aureus* ATCC 25923	37.5 ^a^	37.5 ^a^
	*E. coli* ATCC 25922	300 ^b^	300 ^b^
PE2	*S. aureus* ATCC 25923	18.75 ^c^	18.75 ^c^
	*E. coli* ATCC 25922	133 ^d^	133 ^d^

PE1: propolis extract 1, PE2: propolis extract 2, MIC: minimum inhibitory concentration, MBC: minimum bactericidal concentration. Values are identical across three independent experiments. The values within a column followed by different letters (a–d) are significantly different according to the one-way ANOVA test (*p* < 0.001). For both tested samples and strains, MIC values were identical to MBC values. *S. aureus*: *Staphylococcus aureus*; *E. coli*: *Escherichia coli*.

**Table 5:** The docking results for the bioactive components of Algerian propolis extract and ML334 with KEAP1 (PDB ID: 4L7B).

Complex (4L7B–Compound)	Binding Affinity (kcal/mol)	Hydrogen Bonds	Distance (Å)	Hydrophobic Interactions	Distance (Å)
1,3-*O*-Caffeoyl-dihydrocaffeoylglycerol	−11.020	Asn414, Gln530, Ser602, Ser555, Asn414, Val463, Ile416, Gly462, Tyr525	[1.81–2.99]	Tyr334, Arg415, Ile416, Ala556,	[4.63–5.15]
Ferulic acid 4-*O*-glucoside	−10.021	Ser602, Ile416, Val463, Leu557, Val604, Gly462, Gly509, Ala556	[1.61–2.81]	Arg415, Ala556	[2.83–3.45]
Quercetin 3-methyl ether	−8.991	Ser363, Asn414, Asn382, Ser555, Gly364,	[1.72–2.79]	Arg415, Ala556	[5.21–5.42]
Pinobanksin	−8.379	Gly364, Ile416, Ala510, Arg415	[1.64–2.99]	Ala336, Arg415, Ala556	[3.62–5.47]
Quercetin 3-*O*-rhamnoside	−8.335	Ser363, Gln530, Ser508, Asn414, Tyr334	[1.91–2.82]	Tyr572, Ala556, Arg415	[4.34–4.89]
Caffeic acid phenylethyl ester	−7.180	Arg415, Leu557, Leu365, Gly462	[1.83–2.78]	Ala556	[4.09]
Gallic acid 4-*O*-glucoside	−6.971	Ser602, Asn382, Arg415, Gly462, Gly509	[1.83–3.10]	Arg415, Ala556	[3.59–4.77]
Hesperetin	−6.990	Ser363, Gln530, Ser508, Asn414, Tyr525	[1.95–2.68]	Ala556	[4.87]
Caffeic acid cinnamyl ester	−7.009	Arg415, Leu365, Ile416, Gly603, Ser602	[1.88–2.97]	Gly364, Leu365, Arg415, Ile416, Ala556,	[4.83–5.03]
ML334 (reference ligand)	−6.807	Ser363, Asn414, Arg415, Ser602, Arg380	[1.71–3.04]	Arg415, Ala556, Tyr334	[3.48–4.85]

## Data Availability

The authors declare that the data supporting the findings of this study are available within the paper and its Supplemental Material.

## Supplementary Materials

The following supporting information can be downloaded at: https://www.mdpi.com/article/10.3390/cimb47090761/s1.

## Abbreviations

ABTS	2:2′-azino-bis (3-ethylbenzothiazoline-6-sulfonic acid)
ADME-Tox	absorption, distribution, metabolism, excretion, and toxicology
DPPH	2,2-diphenyl-1-picrylhydrazyl
FRAP	ferric reducing antioxidant power
GC-MS	gas chromatography–mass spectrometry
PCA	principal component analysis
PDB	Protein Data Bank
PE1	propolis extract 1
PE2	propolis extract 2
Phen	1,10-phenanthroline
RMSD	root mean square deviation
TFC	total flavonoid content
TPC	total phenolic content
UHPLC-DAD-ESI/MS	ultra-high-performance liquid chromatography with diode array detection and electrospray ionization mass spectrometry
