# Supplementary material for: Comparative Molecular Profiling and Bioactivity Analysis of Algerian Propolis: Antioxidant, Antibacterial Activities, and In Silico NRF2-KEAP1 Pathway Modulation
Source: Curr Issues Mol Biol. 2025 Sep 15;47(9):761. doi: 10.3390/cimb47090761 (PMC12468889; doi:10.3390/cimb47090761)
Supplement: Supplementary file 1 [file cimb-47-00761-s001.zip › cimb-3846306-Supplementary.pdf]

SUPPLEMENTARTY DOCUMENT

For

Comparative Molecular Profiling and Bioactivity Analysis of Algerian Propolis: Antioxidant, Antibacterial Activities, and In Silico NRF2-KEAP1 Pathway Modulation

Amel Reguig [1], Ahmed Messai [2], Ibtissem Kahina Bedaida [3], Diana C. G. A. Pinto [4], Chawki Bensouici[5], Abdelmoneim Tarek Ouamane [6], Artur M. S. Silva [4] and Jean-Philippe Roy [7,*]

Contents:

Figure S1. Cartographic representation of Algerian propolis sample collecting regions.

Figure S2. Total Ion Chromatogram (TIC) of Algerian Propolis Extracts (A) Propolis Extract 1 (PE1), (B) Propolis Extract 2 (PE2), (TIC/min).

Figure S3. Ultra-High Performance Liquid Chromatography with Diode Array Detection and Electrospray Ionization Mass Spectrometry (UHPLC-DAD-ESI/MS) Chromatographic visualization of Algerian propolis extracts (C) Propolis Extract 1 (PE1), (D) Propolis Extract 2 (PE2).

Figure S4. Principal component analysis (PCA) biplot illustrating the distribution of bioactive compounds identified in propolis extracts through ultra-high performance liquid chromatography with diode array detection and electrospray ionization mass spectrometry.

Table S1. Geographic characteristics of the Algerian propolis sample collection sites.

Table S2. Absorption, Distribution, Metabolism, Excretion, and Toxicology (ADME-Tox) properties of bioactive compounds from Algerian propolis extracts and their drug-like attribute.

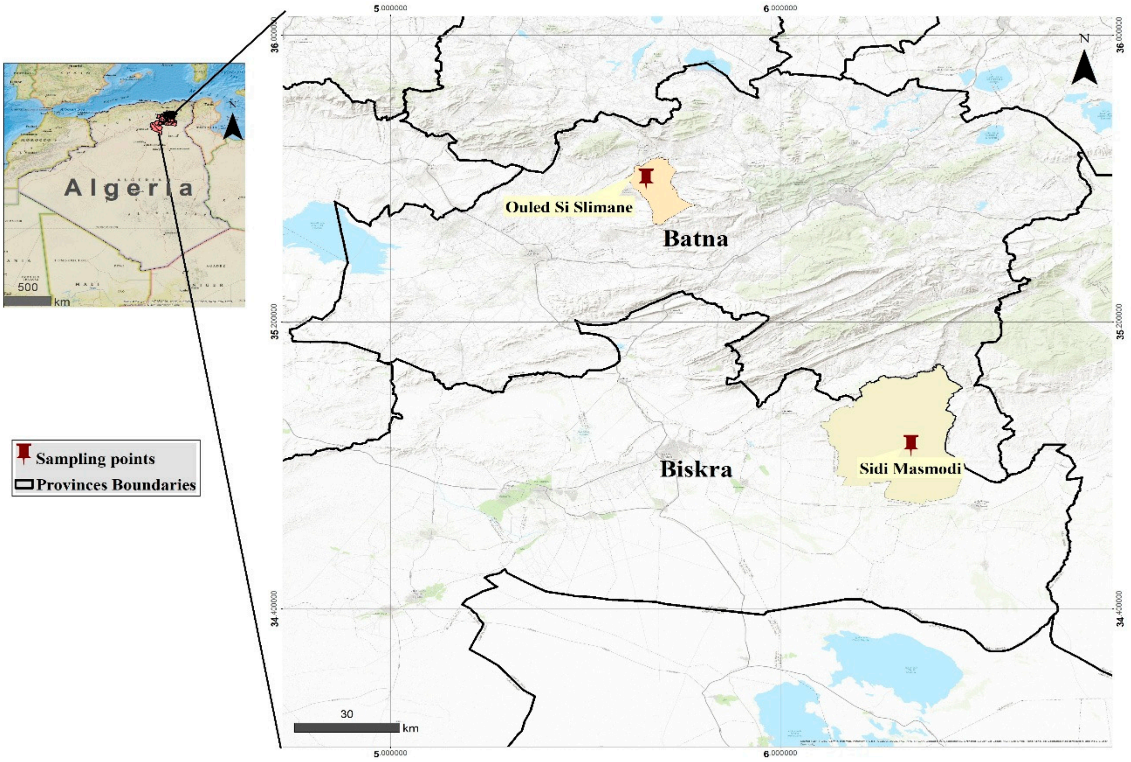

Figure S1. Cartographic representation of Algerian propolis sample collecting regions.

Table S1. Geographic characteristics of the Algerian propolis sample collection sites.

Sample	Site name	Latitude (N)	Longitude (W)	Altitude (m)	Climate	Temp (°C / °F)	Pluviometry (mm / inch)
P1	Ouled Si-Slimane (Batna)	35°36'39"	5°37'58"	1045	Cfa	13.5 / 56.4	496 / 19.5
P2	Sidi Masmodi (Biskra)	34°51'10.5"	6°18'51.5"	121	BWh	21.1 / 69.9	125 / 4.9

According to the Köppen climate classification, Cfa is a humid subtropical climate and BWh is a hot desert climate. The data were obtained during the year 2020.

A.

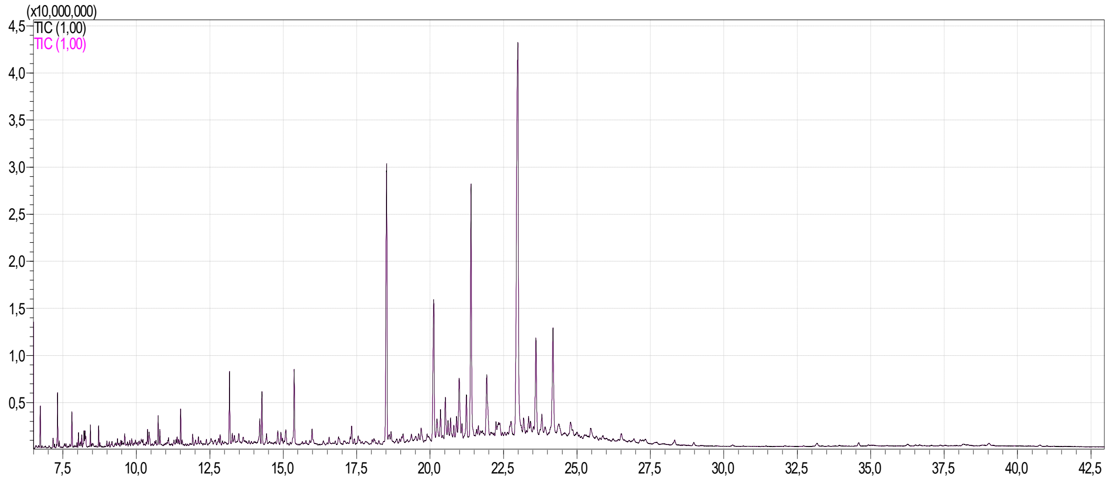

B.

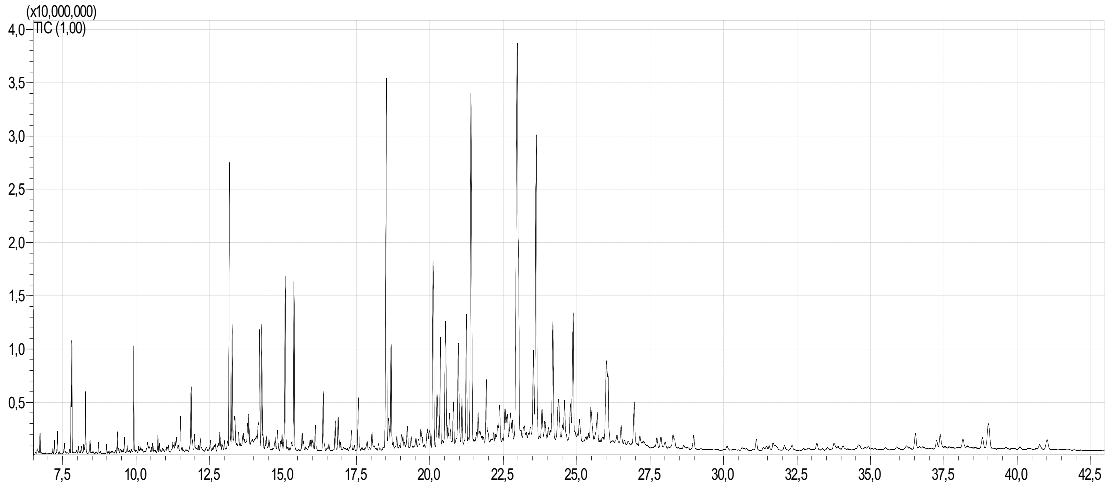

Figure S2. Total Ion Chromatogram (TIC) of Algerian Propolis Extracts (A) Propolis Extract 1 (**PE1)**, (B) Propolis Extract 2 (**PE2)**, (TIC/min).

C.

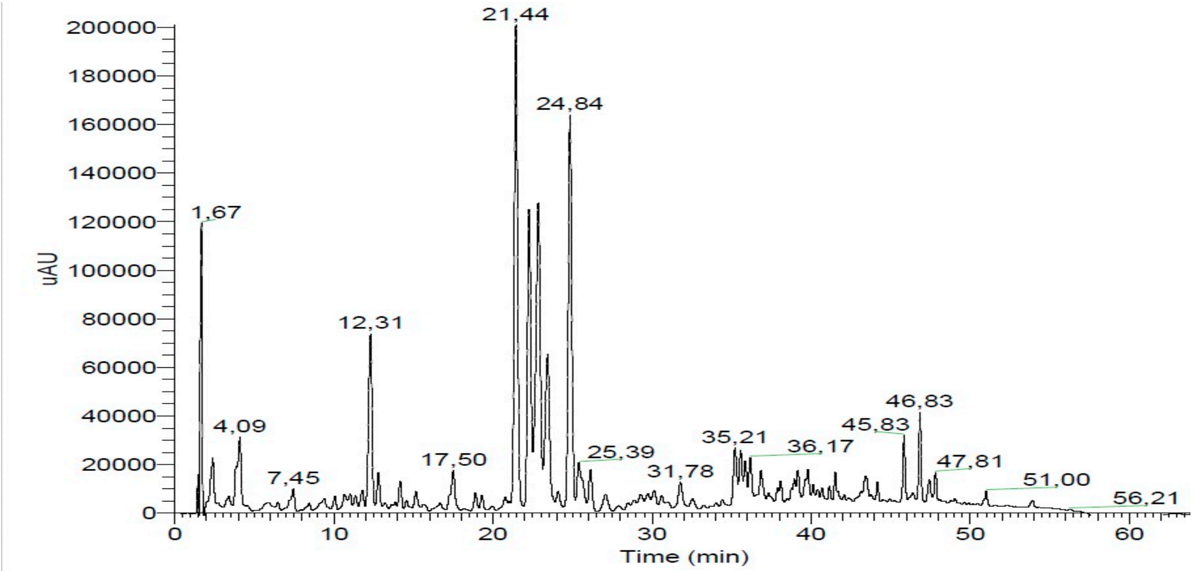

D.

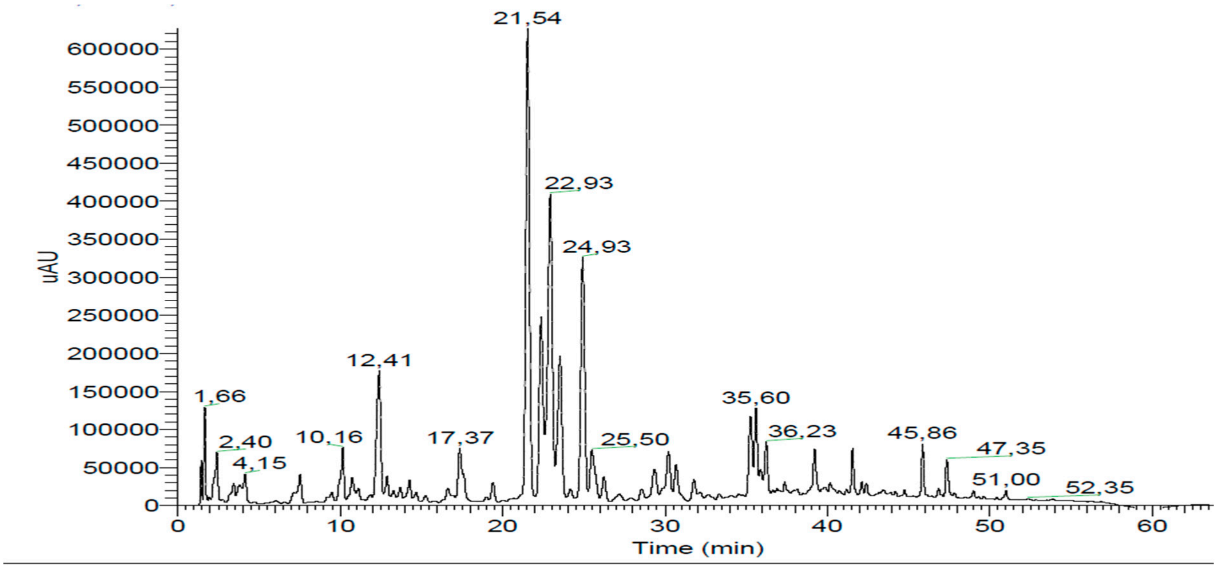

Figure S3. Ultra-High Performance Liquid Chromatography with Diode Array Detection and Electrospray Ionization Mass Spectrometry (UHPLC-DAD-ESI/MS) Chromatographic visualization of Algerian propolis extracts (C) Propolis Extract 1 (**PE1)**, (D) Propolis Extract 2 (**PE2)**.

Table S2. Absorption, Distribution, Metabolism, Excretion, and Toxicology (ADME-Tox) properties of bioactive compounds from Algerian propolis extracts and their drug-like attributes.

									Toxicity	
**Compounds**	**MW (g/mol)**	**Consensus Log P**	**Log S (ESOL)**	**GI absorption**	**CYP450 inhibition**	**Lipinski**	**Bioavailability Score**	**Synthetic accessibility**	**Predicted LD_{50} (mg/kg)**	**Predicted Toxicity Class**
1,3-*O*-Caffeoyl dihydrocaffeoylglycerol	272.25	1.84	-3.49	High	No	Yes	0.55	3.01	2000	4
Ferulic acid 4-*O*-glucoside	355.32	-0.89	-1.14	Low	No	Yes	0.56	4.46	4000	5
Quercetin 3-methyl ether	316.26	1.75	-3.89	High	Yes	Yes	0.55	3.29	5000	5
Pinobanksin	285.27	1.63	-2.52	High	No	Yes	0.55	3.34	2500	5
Quercetin 3-*O*-rhamnoside	448.38	0.26	-3.33	Low	No	No	0.17	5.28	5000	5
Caffeic acid phenylethyl ester	284.31	3.09	-4.24	High	No	Yes	0.55	2.64	5000	5
Gallic acid 4-*O*-glucoside	331.25	-1.87	-0.76	Low	No	Yes	0.11	4.13	3750	5
Hesperetin	304.29	1.61	-3.10	High	No	Yes	0.55	4.02	435	4
Caffeic acid cinnamyl ester	296.32	3.21	-3.96	High	No	Yes	0.55	2.62	1520	4
ML334 (Reference ligand)	459.51	2.52	-4.49	High	No	Yes	0.56	4.10	210	3

MW: Molecular weight, Log P: Octanol-water partition coefficient (measure of lipophilicity), Log S (ESOL): Estimated water solubility, GI absorption: Gastrointestinal absorption, CYP450 inhibition: Inhibition of cytochrome P450 enzymes, LD_{50}: median lethal dose.

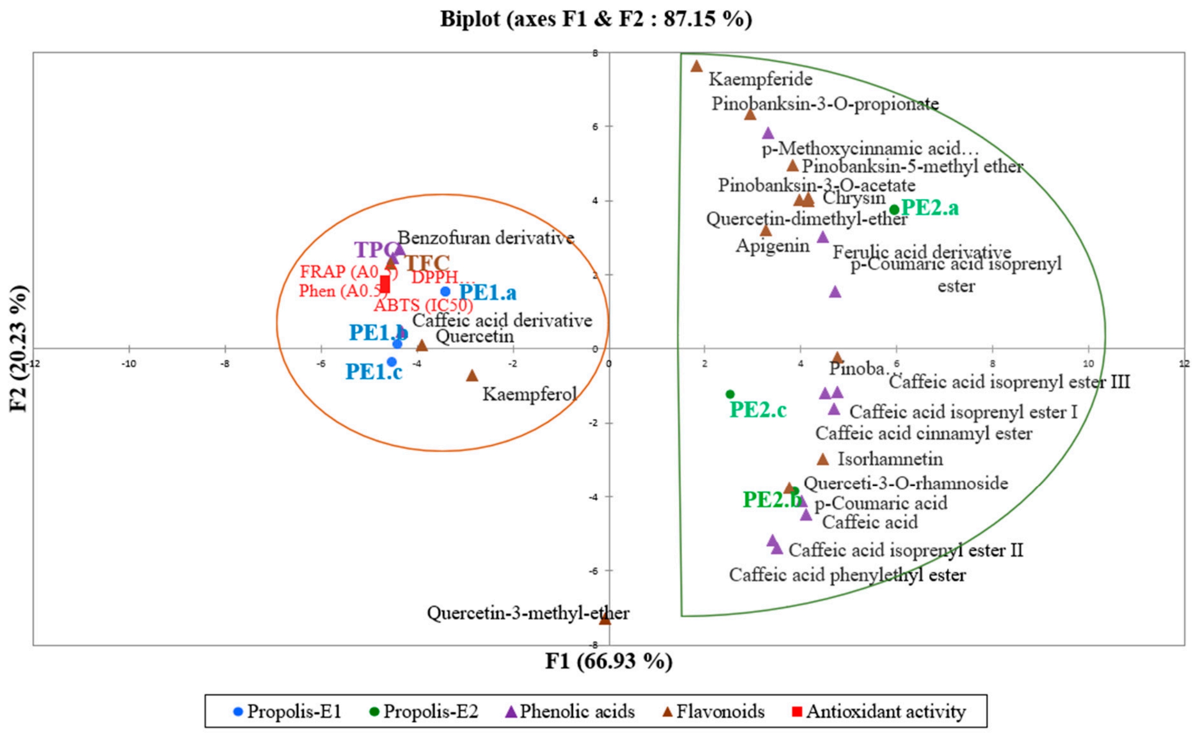

TPC: Total phenolic content, TFC: Total flavonoid content, DPPH: 2,2-Diphenyl-1-picrylhydrazyl, ABTS: 2,2'-azino-bis (3-ethylbenzothiazoline-6-sulfonic acid), FRAP: Ferric reducing antioxidant power, Phen: 1,10-Phenanthroline, IC_{50}: 50% inhibitory concentration, $PE._1$, $PE._2$, $PE._3$: Propolis extract 1(or 2) (three independent replicates).

Figure S4. Principal component analysis (PCA) biplot illustrating the distribution of bioactive compounds identified in propolis extracts through ultra-high performance liquid chromatography with diode array detection and electrospray ionization mass spectrometry.
